# Two-Way Shear Resistance of FRP Reinforced-Concrete Slabs: Data and a Comparative Study

**DOI:** 10.3390/polym14183799

**Published:** 2022-09-11

**Authors:** Fahid Aslam, Mohamed AbdelMongy, Majed Alzara, Taha Ibrahim, Ahmed Farouk Deifalla, Ahmed M. Yosri

**Affiliations:** 1 Department of civil Engineering, College of Engineering in Al-kharj, Prince Sattam bin Abdulaziz University, Al-Kharj 16278, Saudi Arabia; 2 Department of Civil Engineering, College of Engineering, Jouf University, Sakakah 72388, Saudi Arabia; 3 Civil Engineering Department, Faculty of Engineering, Al-Azhar University, Cairo 11651, Egypt; 4 Department of Structural Engineering, Shoubra Faculty of Engineering, Benha University, Cairo 11629, Egypt; 5 Department of Structural Engineering and Construction Management, Future University in Egypt, New Cairo City 11835, Egypt

**Keywords:** design, two-way shear, GFRP, CFRP, FRP, BFRP

## Abstract

This study aims to investigate the two-way shear strength of concrete slabs with FRP reinforcements. Twenty-one strength models were briefly outlined and compared. In addition, information on a total of 248 concrete slabs with FRP reinforcements were collected from 50 different research studies. Moreover, behavior trends and correlations between their strength and various parameters were identified and discussed. Strength models were compared to each other with respect to the experimentally measured strength, which were conducted by comparing overall performance versus selected basic variables. Areas of future research were identified. Concluding remarks were outlined and discussed, which could help further the development of future design codes. The ACI is the least consistent model because it does not include the effects of size, dowel action, and depth-to-control perimeter ratio. While the EE-b is the most consistent model with respect to the size effect, concrete compressive strength, depth to control perimeter ratio, and the shear span-to-depth ratio. This is because of it using experimentally observed behavior as well as being based on mechanical bases.

## 1. Introduction

**Figure 1:**
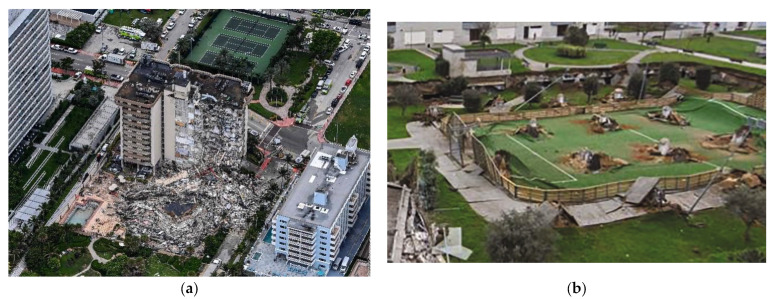
Collapsed of (**a**) condominium building [1] and (**b**) a parking garage on a playground [2].

In 2021, victims of the collapse of a condominium building [1] that is shown in Figure 1(a) totaled 98 people. In addition, a parking garage collapsed suddenly on a playground in Spain [2], as shown in Figure 1(b). Moreover, most of the two-way shear designs of reinforced concrete (RC) slabs are empirical or semi-empirical. Thus, extensive research efforts are direct towards understanding the two-way shear types. However, the mechanism of the two-way shear of the slabs is complicated; thus, it is still open for investigation [3,4,5,6]. The two-way shear resistance of concrete slabs that are without shear reinforcements is composed of several resistance mechanisms, as follows: (1) flexure reinforcements resist shear through using dowel shear; (2) aggregates resist shear across the sides of the diagonal concrete crack through using aggregate interlock and friction; (3) uncracked concrete resists shear through using direct shear [7,8,9].

To avoid corrosion problems, replacing the conventional reinforcement with fiber-reinforced polymers (FRP) reinforcements in concrete slabs is a common solution [10]. In addition, FRP reinforcements are magnetic neutral and have a high strength-to-weight ratio. Thus, they are the best choice to use in buildings that are subjected to severe environmental conditions including, and not limited to, wet-dry cycles, de-icing salts, and freeze-thaw cycles. Many researchers have investigated the behavior of new and existing beams and slabs that are reinforced with FRP bars or fabrics under one-way and two-way shear as well as torsion, mostly through experimental investigations [11,12,13,14,15]. Many research studies have tackled the two-way shear of concrete FRP reinforcements, while very few mechanical models were developed for this case [8]. The FRP’s failure is brittle; thus, before failure, the FRP-reinforced concrete cracks are wider when compared to those in conventional RC [16,17,18]. Wider cracks significantly affect the various mechanisms of the two-way shear strength.

The traditional two-way shear design equations for RC slabs are based on theories that were developed in the early 1960s. These models were based on studies of that period’s tested specimens; however, over the last few decades, much more testing was conducted which show several drawbacks of these methods including, and not limited to, size effect and those models being severely unconservative in many situations. Hence, there is a room for improvements to the two-way shear design models, which could help design code developments [19,20].

This study aims to assess the available methods for study of the two-way shear strength of FRP-reinforced concrete slabs. A state-of-the-art review of design codes, guides, and models for the study of the two-way shear strength of FRP-reinforced concrete slabs was outlined. An extensive review of the experimental testing of the FRP-reinforced concrete slabs that were tested under two-way loads was compiled. The studies that used to extract the models and their experimental testing were collected through various engineering search engines including, and not limited to: Google Scholar, Science Direct, MDPI Hub, and Engineering Village. The strengths calculated using all models were compared with those that were measured by testing. Concluding remarks were outlined and discussed.

## 2. Research Significance

Many researchers have proposed design models that address the two-way shear strength of RC slabs with FRP. Although safety is the main goal for the design purposes, evaluating these design models is a necessity. The accuracy of these models was assessed based on data from a limited experimental database. Thus, this study provides the community with an extensive collection of models and tested specimens as well as a comparison between the accuracy of each of these models. These results can help to improve the code developments.

## 3. Simplified Strength Models

For the study of the two-way shear strength of FRP-RC slabs, several simplified strength models have been proposed, either by modifications for conventional concrete slabs or empirically based on limited test data. The two-way shear design provisions of the North American design codes have neglected the effect of flexure reinforcement on strength. They focused on the direct shear resistance of the compression zone. This could be reasonable for conventional steel reinforcements with relatively high stiffness when compared to the FRP ones. Thus, the direct shear component governs the two-way shear strength. However, due to the relatively lower stiffness of the FRP when compared to the steel one, a dowel action could be a more significant contributor to its strength. Details and the background of various models are outlined in this section. V is the two-way shear failure load. E is the Young’s modulus of the FRP. d is the effective depth. $f_{c}^{\prime }$ is the concrete compressive strength. ρ is the flexure reinforcement ratio. b and c are the column dimensions. A and B are the slab dimensions. $E_{s}$ is the Young’s modulus of the steel. $b_{0.5d}$ is the control perimeter at 0.5d, which is taken as $2\left(b+c+2d\right)$. $b_{1.5d}$ is the control perimeter at 1.5d, which is taken as $2\left(b+c+6d\right)$. $b_{2.0d}$ is the control perimeter at 2.0 d, which is taken as $2\left(b+c+8d\right)$.

### 3.1. Gardner (1990)

In 1990, Gardner developed a strength model, which will be referred to herein as G [21]. It is based on an experimental database for two-way shear, and the existing design codes were assessed. Gardner concluded that considering the size effect and the flexure reinforcement ratio provides a more reliable design model; thus, when fitting it to the experimental database of that time, the two-way shear is calculated such that:

(1) V=1.36 100ρfc′131d14b1.5dd

### 3.2. Japanese Approaches (1997), JSCE

In 1997, the JSCE [22] used a similar approach to the conventional North American design codes, and they implemented the assumption that the strength was proportional to the square root of the concrete compressive strength. Thus, they implemented the strain approach on the British Standard of that time and proposed that the two-way shear was calculated such that:

(2) $$V=\beta_{d}\beta_{\rho }\beta_{r}f_{Pcd}\frac{1}{\alpha }b_{0.5d}d$$

where βd=1000d14≤1.5, $\beta_{\rho }\sqrt[{3}]{100\rho \frac{E}{E_{s}}}=\leq 1.5$, βr=1+11+0.25b0.5dd, $f_{\mathrm{Pcd}}=0.2\sqrt{f_{c}^{\prime }}\;\leq 1.2$, $\alpha =1+1.5\left[\frac{e_{x}+e_{y}}{\sqrt{\mathrm{AB}}}\right]$, and $e_{x}$ and $e_{y}$ are the loading eccentricity in the x and y direction of the slabs, respectively.

### 3.3. Elghandour (2000), EG [23]

In 2000, Elghandour developed a strength model, which will be referred to as EG [23]. The model was developed using the strain and stress approaches to determine the steel area equivalent to the FRP area, and it can be used in the conventional two-way shear models. Thus, they implemented the strain approach, with a limit of the value of 0.0045 for the strain and the British Standard of the time, and proposed that the two-way shear was calculated such that:

(3) V=0.79fc′2013100ρEEs1.813400d14b1.5dd

### 3.4. Mattys and Taerwe (2000), MT

In 2000, Mattys and Taerwe, developed a strength model, which will be referred to herein as MT [24]. It was developed based on the observed behavior of the experimental testing of FRP-reinforced concrete slabs; their stiffness is less than that of conventional reinforced slabs. In addition, the depth and axial stiffness of FRP reinforcements have a significant effect on their strength; thus, they modified the British design code to be as follows:

(4) V=1.36100ρfc′EEs131d14b1.5dd

### 3.5. Ospina (2003), O

In 2003, Ospina [25] developed a model (O), which is based on their experimental observations, and it was found that the strength is affected by the axial stiffness of the FRP reinforcements and the bond they have with the concrete. Thus, when it is modified, the MT model is expressed as follows:

(5) V=2.77ρfc′13 EEsb1.5dd

### 3.6. Zaghloul (2003), Z

Zaghloul [26] has adapted the one-way shear design of the FRP-reinforced concrete of the Canadian design codes and multiplied it by a factor of two and introduced a perimeter size effect factor, such that:

(6) V=0.07Eρfc′130.44+5.16αsdb0.5d b0.5dd

$\alpha_{s}$ = 4, 3, and 2 for an inner connection, an edge connection, and a corner connection, respectively. 

### 3.7. Jacbson (2005), Jb [27]

This is an empirical model which was developed through experimental testing.

(7) V=4.5 ρfc′121d14b1.5dd 

### 3.8. ACI (2005), ACI [28]

This ACI model accounts for the effect of the direct shear of the compression zone of the concrete, where the ACI equation for the conventionally reinforced concrete slabs ($0.3\sqrt{f_{c}^{\prime }}db_{0.5d}$) is multiplied by the factor (2.5 k). Thus, the shear strength is calculated such that:

(8) $$V=0.8\sqrt{f_{c}^{\prime }}kdb_{0.5d}$$

where $k=\sqrt{2\rho n+{\left(\rho n\right)}^{2}}-\rho n$, modular ratio ($n)=\frac{E}{E_{c}}$, concrete young’s modulus, and $E_{c}=4750\sqrt{f_{c}^{\prime }}$.

### 3.9. Elgamal (2005), E [29]

Elgamal developed a model, which was based on the experimentally observed fact that the two-way shear is affected by the FRP axial stiffness, and the slab end conditions are in terms of their continuity or them having an edge beam. Thus, the strength was proposed, such that:

(9) V=0.33fc′20.62Eρ131+8db0.5d1.2Nb0.5dd

where *n* = 0, 1, and 2 represents a simple slab, a continuous, one-sided slab, and a continuous, two-sided slab, respectively.

### 3.10. Zhang (2006), Zg [30,31]

Zhang developed a design model which included the following assumptions: (1) that two-way shear failure occurs after the critical diagonal shear crack passes through the compression zone; (2) that failure is related to concrete tensile strength; (3) that dowel action contributes to the strength. In addition, the model was calibrated using the experimental database that was available at that time, such that:

(10) V=⌊0.25+1.10 100ρEEs12⌋1d1/5fc′13b1.5dd

### 3.11. Theodorkopoulos and Swamy (2008), TS [32]

The proposed model was based on moment–shear interaction, which determined the compression zone depth using the tensile elastic stiffness of the FRP reinforcements and the bond between FRP reinforcements and the concrete.

(11) V=0.117fc′0.823100d162αfλf1+αfλfb0.5dd

where λf=0.556−1+1+48αf≤1, $\alpha_{f}=0.058\frac{\rho E}{f_{c}^{\prime }}\geq 0.33$.

### 3.12. CSA-S806-12 (2012), CSA [33]

The design model was developed, based on the conventional concrete design code, however, it was modified for FRP reinforcements instead of steel ones.

(12) V=b0.5dd0.0281+2βcEρfc′130.147Eρfc′130.19+αsdb0.5d0.056Eρfc′13

$\beta_{c}=$ ratio between the long and short side of the loading area; $\alpha_{s}$ = 4, 3, and 2 for an inner connection, an edge connection, and a corner connection, respectively. $b_{0.5d}=4\left(c+d\right)$.

### 3.13. Nguyen and Rovnak (2013), NR [34]

A fracture mechanics-based semi-empirical model was developed, which considered the effects of the following: (1) span-to-depth ratio; (2) the effective depth; (3) the dowel action.

(13) $$V=\sqrt{\frac{400}{d}}\left[\frac{0.8d}{a}\right]{\left(\rho /100\right)}^{0.33}E^{0.33}f_{c}^{\prime }{}^{0.3}b_{0.5d}$$

where a is the slab’s shear span.

### 3.14. Hassan, et al. (2017), H [35]

The model is a modification of the CSA which combines the three equations into a single formula. Then, it used a multi-linear regression technique to fit the 69 specimens in the experimental database using a power equation, such that:

(14) V=0.0650.65+4db0.5dEρfc′13125d16b0.5dd

### 3.15. Kara and Sinani (2017), KS [36]

The KS model is a modification of the MT model that replaces the coefficient with 0.46 instead of 1.36 and removes the d parameter, such that:

(15) V=0.46100ρfc′EEs13b1.5dd

### 3.16. Oller, et al. (2018), CCCM [37]

The CCCM model was developed, based on the model by Mari and co-workers [38], and it is a unified model for two-way shear; thus, it applies the following assumptions: (1) the strains are higher due to the lower modulus of elasticity of the FRP bars; (2) the cracks are wider; (3) the basic perimeter at the point of failure is lower in an FRP-reinforced concrete (RC) slab than it is in a conventional RC slab. Thus, the shear capacity is calculated such that:

(16) V=ξXd2.5fctβb0.5dd≥0.25fc′231.8ξKc+20dob0.5dd

where $\xi =\frac{2}{\sqrt{1+\frac{d}{200}}}\left(\frac{d}{a}\right){\;}^{0.2}\leq 0.45$, Xd=0.75αeρ13, fct=0.3fc′23≤4.60 MPa, $d_{o}=d\geq \;100\;mm$, $\alpha_{e}=\frac{E}{E_{c}}$, $K_{c}=\frac{X}{d}\leq 0.2$, Ec=22000fc′100.3≤39 GPa.

### 3.17. Hemzah, et al. (2019), Hz [39]

Using numerical modeling and an experimental database, a two-way shear formula which considers the flexure reinforcement ratio and type, the compressive strength of concrete, and the shape of the column was developed, such that:

(17) V=13fc′12k90fc′0.335ρ0.39EEs0.3b0.5dd

k=0.77 and 0.55  for circular and rectangular columns, respectively.

### 3.18. Elgendy and Elsalakawy (2020), EE [40]

Considering the eccentricity of the slab-column joint, the H model and the EG model were modified, such that:

(18) V=0.33fc′120.62Eρ131+2αsdb0.5d1.2Nb0.5dd

(19) V=0.0650.65+αsdb0.5dEρfc′13125d16b0.5dd

$\alpha_{s}$ = 4, 3, and 2 for an inner connection, an edge connection, and a corner connection, respectively. N = 1, 2, and 3 for a simple slab, a continuous, one-sided slab, and a continuous, two-sided slab, respectively.

### 3.19. Ju, et al. (2021), Ju [41]

To guarantee the lowest probability of failure, the design strength was calculated with the probabilistic method with 95% confidence; thus, the Monte Carlo Simulation (MCS) was used to develop the probability distribution with key uncertain factors, such that:

(20) V=2.3 100ρEEsfc′12db0.5d12b0.5dd

### 3.20. Alrudaini (2022), A [42]

A rational model is developed, which considers the following: (1) concrete compressive strength, elastic properties of reinforcement, reinforcement ratio, and slab depth to the effective perimeter. Each parameter was fitted to the measured strength, such that:

(21) V=0.41 ρEfc′13db0.5d15b0.5dd

Table 1 shows a comparison between the various design models, where it is clear that there is a lack of agreement among researchers regarding the considered parameters and methodology used to account for it. All design methods included the effect of concrete compressive strength in terms of fc′13 or fc′12. Most of the methods included the dowel action in terms of the flexure reinforcement, which was taken as ρ13 or ρ12. More than half of the methods included the FRP type in terms of Young’s modulus, which was taken as E13., or E12. About half of the methods included the size effect in terms of 1d14, 1d15, 1d12, or 21+d200 and included the ratio between the critical perimeter and the depth in terms of 0.44+20.8db0.5d, 1+8db0.5d, 0.19+4db0.5d, 0.65+4db0.5d, 1+8db0.5d, 0.65+4db0.5d, db0.5d12, or db0.5d15. On the other hand, very few models included the compression zone and the shear span-to-depth ratio.

## 4. Experimental Database Profile

**Figure 2:**
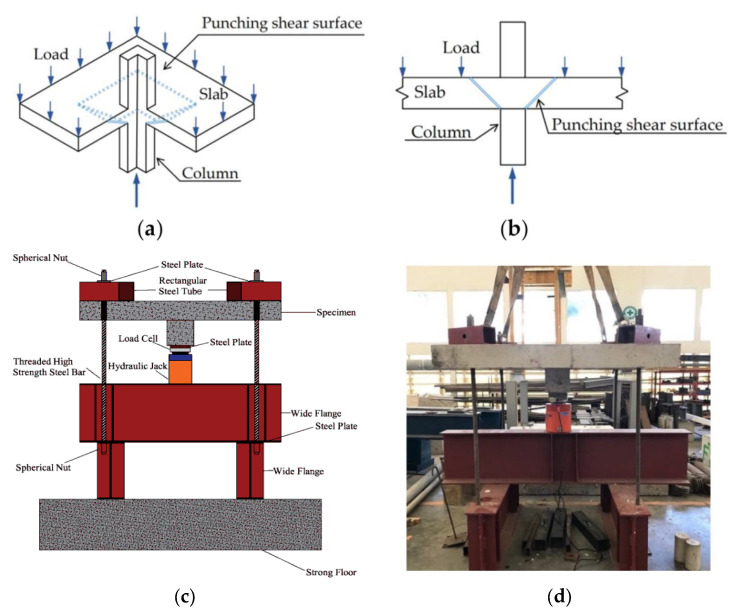
Two-way shear (**a**) failure isometric view; (**b**) failure elevation; (**c**) loading test setup schematic; (**d**) actual loading test setup [43,44].

Over the last 30 years, a significant number of experimentally tested specimens failed due to the effect of two-way shear. The most comprehensive experimental database, when compared to those of previous studies [2,39,41,43], was produced with a total of 248 slabs with FRP reinforcements that were collected from 50 different research studies. All the gathered slabs were subjected to two-way shear loading and failed, suddenly, under the application of two-way shear, as shown in Figure 2.

**Figure 3:**
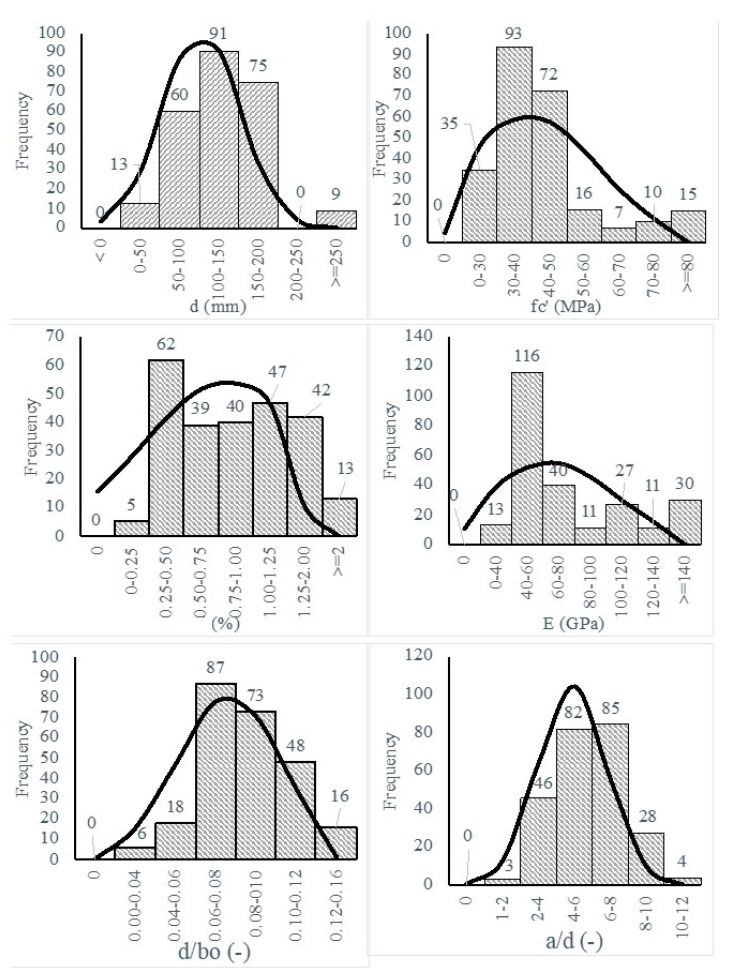
Frequencies and ranges of the tested column-slab connections with FRP.

Table 2 shows a detailed description of the experimental database, where E is the Young’s modulus of FRP, d is the effective depth, $f_{c}^{\prime }$ is the concrete compressive strength, ρ is the flexure reinforcement ratio, b and c are the column dimensions, A and B are the slab dimensions, and FRP type including carbon FRP (CFRP), glass FRP (GFRP), and Basalt FRP (BFRP) are listed. Although FRP reinforcements could have several shapes and configurations, these variations were considered in terms of ρ and E. Figure 3 shows the frequency and the range of each variable. All variables cover a wide range of values, while also being normally distributed.

## 5. Behavior Patterns

**Figure 4:**
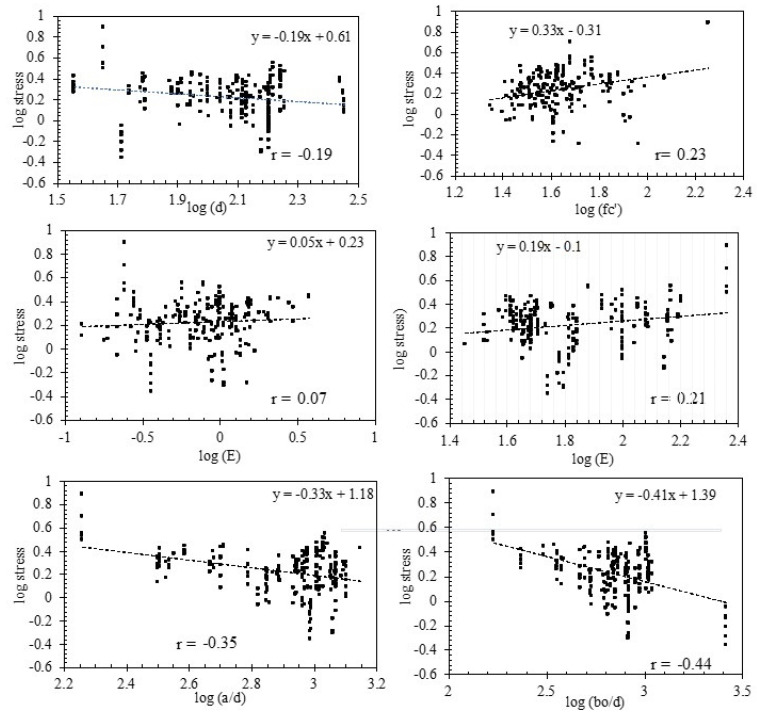
Stress versus basic variables.

Based on the existing models and previous studies of slabs, the relationship between the shear stress (V/b_o_d) and the effective parameters including d, E, ρ, fc’, d/b_o_, and a/d is a power relationship. Thus, Figure 4 shows the scatter plots for the pattern of the log of the shear stress versus the log of the effective parameters. The scatter plots do not allow a straightforward interpretation of the data because of the significant dispersion and poor distribution of the test parameters; thus, the best regression fit line and the Pearson correlation coefficients (r) are shown in Figure 4. The inclination of the best fit lines between the stress and basic variables d, E, ρ, fc’, d/b_o_, and a/d were the values of −0.19, 0.19, 0.05, 0.34, 0.33, and 0.41, respectively. Comparing these values to those that were used in selected models, as shown in Table 1, it can be shown that variables ρ and fc’ have quite similar power coefficients, while E, d/b_o_, and a/d are significantly different, and d is only like that of one selected model.

## 6. Pearson Correlation

The correlation coefficients between the shear stress and the basic variables d, E, ρ, fc’, d/b_o_, and a/d were calculated, as shown in Figure 4, where their values are −0.19, 0.21, 0.07, 0.23, −0.34, and −0.43, respectively. Therefore, the evidence is sufficient to say that the shear strength is correlated to the basic variables in a reasonable manner, except for the flexure reinforcement ratio. This could be because the effect of the flexure reinforcement varies significantly based on its value [83]. Since the experimental database covered a wide range of flexure reinforcement ratio values, it provided a misleading value for the correlation coefficient.

## 7. Comparison between Selected Models

**Figure 5:**
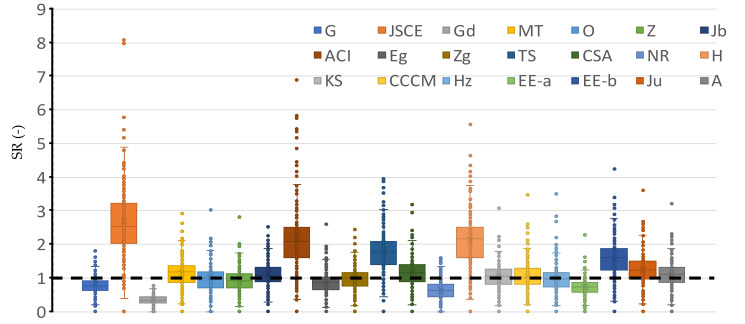
The performance of selected models.

All collected models were used to calculate the strengths of the slab column connections that were in the experimental database. Three categories of comparison were defined: graphical, central tendency, and statistical goodness-of-fit. The ratio between the measured and calculated strength was taken as the safety ratio (SR). An SR value that is close to unity means that the prediction is accurate. An SR value that is more than the unity indicates that the prediction is conservative. An SR value that is less than the unity mean that the shear strength was overestimated and so, the prediction is conservative. Statistical measures in terms of the coefficient of determination (R^2^), the root mean square error (RMSE), the mean average error (MAE), the mean, the coefficient of variation (C.O.V.), the lower value with a 95% confidence level (Lower 95%), the maximum value, and minimum value were applied on the SR for each selected model, as shown in Table 3 and Figure 5. Table 3 shows central tendency and statistical goodness-of-fit for all the selected models, which is helpful for the future development of the design models. The JSCE, the ACI, and the H models are over-conservative, with an average value of 2.71, 2.18, and 2.16, respectively. The Zg, the EE-b, the Ju, and the A models are more consistent with respect to other models, where the coefficient of variation values of these were 35%, 35%, 36%, and 36%, respectively.

**Figure 6:**
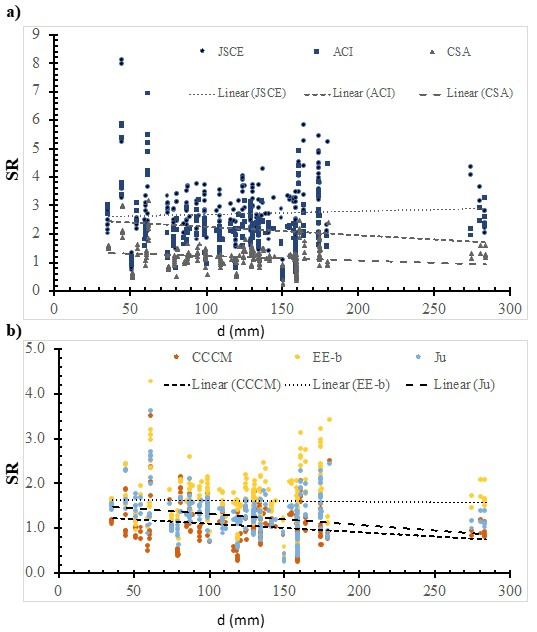
The effect of depth on the SR value, calculated using selected models. (**a**) Design codes; (**b**) Models.

Figure 5 shows a Box plot for all the selected models. A large dispersion and extreme values are observed in the ACI model. Also, severely un-conservative predictions resulted from the application of the Gd and NR models. The recent models (i.e., Ju, A, Hz, and EE-a) provide accurate predictions for the strength (when the mean is close to the unity), as shown in Figure 6. However, the consistency in these models is still lacking (i.e., C.O.V. is higher than 35%), as shown in Table 3. Models considering basic variables in a power form equation seem to be the most accurate and consistent when they are compared with the mechanically based model (CCCM) or the fracture-based model (NR). In addition, from Figure 5, it is clear that each method was developed or calibrated for a nonsystematic margin of safety which was defined by the judgment and experience of each developer(s). This should be managed by a reliability assessment that includes the resistance and load uncertainty. Although it is an interesting topic, it is not in the scope of this study, but it can be the subject of further studies. Moreover, there is a need for further improved mechanically based models that make physical sense, while being simple in their design.

**Figure 7:**
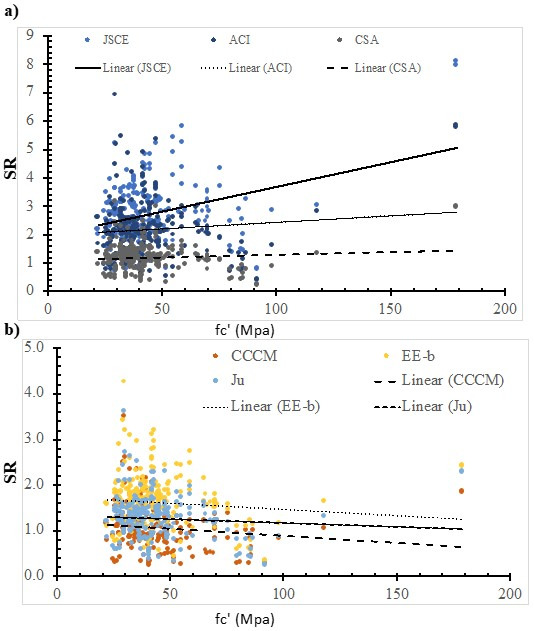
The effect of the concrete compressive strength on the SR value, calculated using selected models. (**a**) Design codes; (**b**) Models.

**Figure 8:**
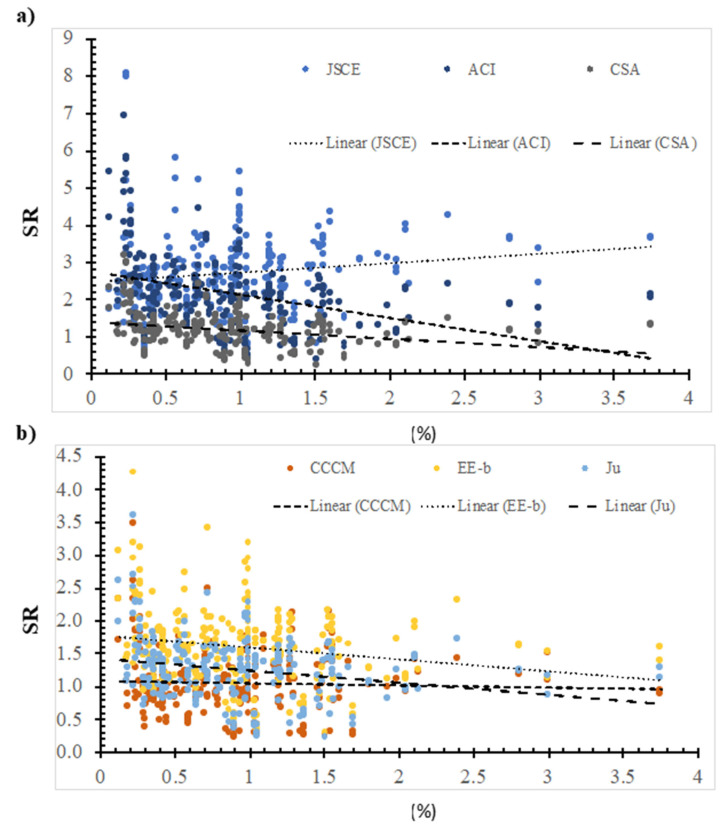
The effect of the flexure reinforcement ratio on the SR value, calculated using selected models. (**a**) Design codes; (**b**) Models.

**Figure 9:**
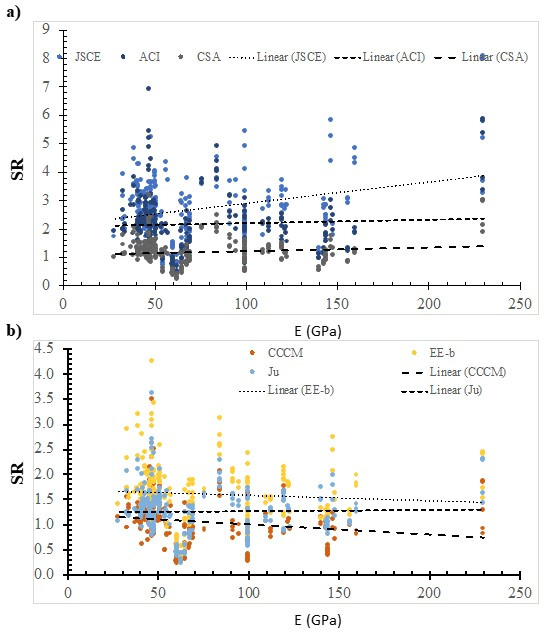
The effect of the Young’s modulus on the SR value, calculated using selected models. (**a**) Design codes; (**b**) Models.

**Figure 10:**
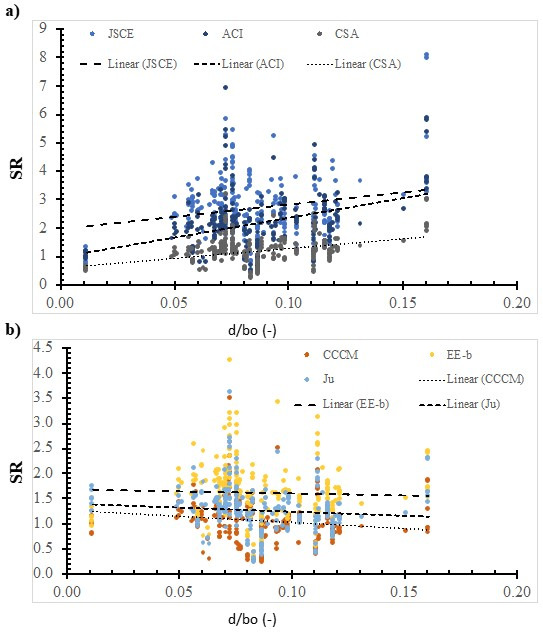
The effect of the ratio between the control perimeter and depth on the SR value, calculated using selected models. (**a**) Design codes; (**b**) Models.

**Figure 11:**
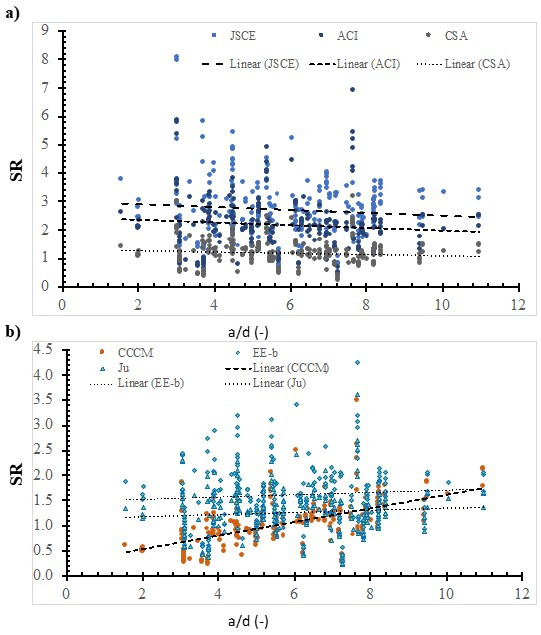
The effect of the shear span-to-depth ratio on the SR value, calculated using selected models. (**a**) Design codes; (**b**) Models.

Figure 5, Figure 6, Figure 7, Figure 8, Figure 9, Figure 10 and Figure 11 shows the SR value, which was calculated using different models, versus the value of the selected effective variable. Although the SR value is affected by the values of the various variables and not only the specific variable in the figure, it is assumed that the presence of the noise, because of the other variables, is insignificant with respect to the specific variable that is in the figure. It is worth noting that this approach was implemented in several pioneering studies as a base for international design codes [11,19,28,33,37,38,42]. In addition, some models do not include that specific variable, however, the experimentally measured strength includes the effect of that variable. Thus, the model’s ability to represent the true value of the strength can be evaluated properly with respect to the effect of that specific variable.

### 7.1. Depth

Figure 6 shows the SR value that was calculated using the ACI model; the JSCE model; the CSA model; the CCCM model; the Ju model; the EE-b model versus the effective depth. In addition, the best fit line was plotted, whose slope was 0.0011, −0.003, −0.0016, −0.0019, −0.0003, and −0.0025 for the JSCE model, the ACI model, the CSA model, the CCCM model, the EE-b model, and the Ju model, respectively. The safety of the selected models decreases with the increase in the depth, except for the JSCE. The best fit line for the SR value that was calculated using the EE-b model is the lowest, thus, it is the most consistent with respect to the depth. However, using the ACI model resulted in the highest SR value, thus, it is the least consistent one. This could be due to the ACI model not having a size effect factor.

### 7.2. Concrete Compressive Strength

Figure 7 shows the SR value that was calculated using the ACI model, the JSCE model, the CSA model, the CCCM model, the Ju model, and the EE-b model versus the concrete compressive strength. In addition, the best fit line was plotted, whose slope was 0.0019, 0.0046, 0.0174, −0.0031, −0.0017, and −0.0027 for the JSCE model, the ACI model, the CSA model, the CCCM model, the EE-b model, and the Ju model, respectively. The safety of the CCCM model, the EE-b model, and the Ju model decreases with the increase in the concrete compressive strength. On the other hand, the safety of the JSCE model, the ACI model, and the CSA model increases with the increase in the concrete compressive strength. The best fit line for the SR value that was calculated using the EE-b model is the lowest; thus, it is the most consistent with respect to the concrete compressive strength. However, using the CSA model resulted in the highest SR value; thus, it is the least consistent one.

### 7.3. Flexure Reinforcment Ratio

Figure 8 shows the SR value that was calculated using the ACI model, the JSCE model, the CSA model, the CCCM model, the Ju model, and the EE-b model versus the flexure reinforcement ratio. In addition, the best fit line was plotted, whose slope was 0.2529, −0.6244, −0.2229, −0.0328, −0.1817, and −0.1844 for the JSCE model, the ACI model, the CSA model, the CCCM model, the EE-b model, and the Ju model, respectively. The safety of the selected models decreases with the increase in flexure reinforcement ratio, except for the JSCE model. The best fit line for the SR value that was calculated using the CCCM model is the lowest; thus, it is the most consistent with respect to the flexure reinforcement ratio. However, using the ACI model resulted in the highest SR value; thus, it is the least consistent one. This could be due to the ACI model not including the flexure reinforcement ratio in the model.

### 7.4. Young’s Modulus

Figure 9 shows the SR value that was calculated using the ACI model, the JSCE model, the CSA model, the CCCM model, the Ju model, and the EE-b model versus the Young’s modulus. In addition, the best fit line was plotted, whose slope was 0.0075, 0.0012, 0.0013, −0.002, −0.0011, and 0.0002 for the JSCE model, the ACI model, the CSA model, the CCCM model, the EE-b model, and the Ju model, respectively. The safety of selected models increases with the increase in Young’s modulus, except for the CCCM model and the EE-b model. The best fit line for the SR value that was calculated using the Ju model is the lowest; thus, it is the most consistent with respect to the Young’s modulus. However, using the JSCE model resulted in the highest SR value; thus, it is the least consistent one.

### 7.5. Depth-to-Control Perimeter Ratio

Figure 10 shows the SR value that was calculated using the ACI model, the JSCE model, the CSA model, the CCCM model, the Ju model, and the EE-b model versus the depth-to-control perimeter ratio. In addition, the best fit line was plotted; whose slope was 8.5935, 13.86, 6.8699, −2.4433, −0.8117, and −1.6327 for the JSCE model, the ACI model, the CSA model, the CCCM model, the EE-b model, and the Ju model, respectively. The safety of the CCCM model, the EE-b model, and the Ju model decreases with the increase in the depth-to-control perimeter ratio. On the other hand, the safety of the JSCE model, the ACI model, and the CSA model increases with the increase in the depth-to-control perimeter ratio. The best fit line for the SR value that was calculated using the EE-b model is the lowest; thus, it is the most consistent with respect to the depth-to-control perimeter ratio. However, using the ACI model resulted in the highest SR value; thus, it is the least consistent one. This could be because the ACI model does not include the effect of this parameter.

### 7.6. Shear Span-to-Depth Ratio

Figure 11 shows the SR value that was calculated using the ACI model, the JSCE model, the CSA model, the CCCM model, the Ju model, and the EE-b model versus the shear span-to-effective depth ratio. In addition, the best fit line was plotted, whose slope was −0.0507, −0.0475, −0.0224, 0.1353, 0.023, and 0.0213 for the JSCE model, the ACI model, the CSA model, the CCCM model, the EE-b model, and the Ju model, respectively. The safety of the JSCE model, the ACI model, the CSA model decreases with the increase in the shear span-to-effective depth ratio. On the other hand, the safety of the CCCM model, the EE-b model, and the Ju model increases with the increase in the shear span-to-effective depth ratio. The best fit line for the SR value that was calculated using the CSA model, the Ju model, and the EE-b model is the lowest; thus, they are the most consistent with respect to the shear span-to-effective depth ratio. However, using the CCCM model resulted in the highest; thus, it is the least consistent one.

## 8. Future Research

Several areas of potential for future research studies were identified as follows:

- Experimental testing of high strength slabs with a compressive strength of more than 45 MPa;
- Experimental testing of ultra-high-performance concrete slabs with a compressive strength of more than 100 MPa;
- Experimental testing of non-slender concrete slabs with a shear span-to-depth ratio of less than 2.5;
- Reliability-based analysis for the safety of the design which includes the variability in the loads, the geometry, the material, and the construction;
- A more reliable and consistent mechanically based model that makes physical sense, while being simple in its design.

## 9. Conclusions

The accuracy of twenty-one selected methods to predict the two-way shear strength of the concrete slabs was assessed. Each method’s ability to predict the two-way strength of concrete slabs without shear reinforcement was studied by comparing predictions against their measured strength from an extensive experimental database comprising a total of 248 slabs from over 50 research studies. Several statistical measures were applied, and the effect of the various basic variables was discussed. The following conclusions were reached:

- The JSCE, the ACI, the H models are over-conservative, with an average value of 2.71, 2.18, and 2.16, respectively. The Zg, the EE-b, the Ju, the A models are more consistent with respect to other models, where the coefficient of variation value was 35%, 35%, 36%, and 36%, respectively.
- The ACI model is the least consistent with respect to the size effect, the dowel action, and the depth-to-control perimeter ratio. This could be due to the fact that the ACI model does not consider these factors in the model.
- The EE-b model is the most consistent with respect to size effect, concrete compressive strength, depth-to-control perimeter ratio, and the shear span-to-depth ratio. This is because of it using experimentally observed behavior as well as it being based on mechanical bases.

## Figures and Tables

**Table 1:** Comparison between design models.

Design Model	Critical Perimeter Location	Size Effect	Dowel Action	Young’s Modulus	Concrete Strength	Control Perimeter-to-Depth Ratio	Compression Zone Depth	Shear Span-to -Depth Ratio
G	1.5 d	d−14	ρ13	------	fc′13	------	------	------
JSCE	0.5 d	d−14	ρ13	E13	fc′12	$\left(1+\frac{1}{1+0.25\frac{b_{0.5d}}{d}}\right)$	------	------
Gd	1.5 d	d−14	ρ13	E13	fc′13	------	------	------
MT	1.5 d	d−14	ρ13	E13	fc′13	------	------	------
O	1.5 d	------	ρ13	E12	fc′13	------	------	------
Z	0.5 d	------	ρ13	E13	fc′13	0.44+20.8db0.5d	------	------
Jb	1.5 d	d−14	ρ12	------	fc′12	------	------	------
ACI	0.5 d	------	------	------	fc′12	------	k	------
EG	0.5 d	------	ρ13	E13	fc′12	1+8db0.5d	------	------
Zg	1.5 d	d−15	ρ12	E12	fc′13	------	------	------
TS	0.5 d	d−16	------	------	fc′23	------	------	------
CSA	0.5 d	------	ρ13	E13	fc′13	0.19+4db0.5d	------	------
NR	0.5 d	d−12	ρ13	E13	fc′13	------	------	ad
H	0.5 d	d−16	ρ13	E13	fc′13	0.65+4db0.5d	------	------
KS	1.5 d	------	ρ13	E13	fc′13	------	------	------
CCCM	0.5 d	2/1+d200	ρ13	E13	fc′23	------	$\frac{X}{d}$	ad−0.2
Hz	0.5 d	------	${\left(\rho \right)}^{0.39}$	${\left(E\right)}^{0;3}$	fc′16	------	------	------
EE-(a)	0.5 d	------	ρ13	E13	fc′12	1+8db0.5d	------	------
EE-(b)	0.5 d	d−16	ρ13	E13	fc′13	0.65+4db0.5d	------	------
Ju	0.5 d	------	ρ12	E12	fc′12	db0.5d12	------	------
A	0.5 d	------	ρ13	E13	fc′13	db0.5d15	------	------

**Table 2:** Experimental database for RC slabs with FRP reinforcements under two-way shear loading.

Year of Study	Specimen Label	A (mm)	B (mm)	b (mm)	c (mm)	d (mm)	fc’ (MPa)	FRP Type	ρ(%)	E (GPa)	V (kN)	#
1993	CFRC-SN1	690	690	75	75	61	42.4	CFRP	0.95	113	93	[45]
	CFRC-SN2	690	690	75	75	61	44.6	CFRP	0.95	113	78	
	CFRC-SN3	690	690	100	100	61	39	CFRP	0.95	113	96	
	CFRC-SN4	690	690	100	100	61	36.6	CFRP	0.95	113	99	
1995	1	600	600	100	100	55	41	CFRP	0.31	100	65	[46]
	2	600	600	100	100	55	52.9	CFRP	0.31	100	61	
	3	600	600	100	100	55	41.5	CFRP	0.31	100	72	
1995	1	1800	1500	250	250	76	30	CFRP	2.05	143	186	[47]
	2	1800	1500	250	250	76	30	CFRP	2.05	143	179	
	3	1800	1500	250	250	76	30	CFRP	1.81	143	199	
	4	1800	1500	250	250	76	30	CFRP	2.05	156	198	
	5	1800	1500	250	250	76	30	CFRP	1.81	156	201	
	6	1800	1500	250	250	76	30	CFRP	1.49	156	190	
1999	1	3000	1800	575	225	175	43	GFRP	1	41.3	500	[48]
	2	3000	1800	575	225	175	43	GFRP	1	41.3	1050	
	3	3000	1800	575	225	175	43	GFRP	1	39.3	875	
	4	3000	1800	575	225	175	43	GFRP	1	39.3	1090	
	5	3000	1800	575	225	175	43	GFRP	1	39.3	1180	
	C1	3000	1800	575	225	175	55	CFRP	1	100	1000	
	C2	3000	1800	575	225	175	55	CFRP	1	100	1200	
	C3	3000	1800	575	225	175	55	CFRP	1	100	1328	
	H2	3000	1800	575	225	175	45	Hybrid	1	160	1055	
	H4	3000	1800	575	225	175	45	Hybrid	1	160	1096	
	H5	3000	1800	575	225	175	45	Hybrid	1	160	1183	
2000	C1	1000	1000	150	150	96	37.3	CFRP	0.27	91.8	181	[24]
	C1’	1000	1000	230	230	95	35.7	CFRP	0.27	91.8	189	
	C2	1000	1000	150	150	95	36.3	CFRP	1.05	95	255	
	C2’	1000	1000	230	230	95	36.3	CFRP	1.05	95	273	
	C3	1000	1000	150	150	126	33.8	CFRP	0.52	92	347	
	C3’	1000	1000	230	230	126	34.3	CFRP	0.52	92	343	
	CS	1000	1000	150	150	95	32.6	CFRP	0.19	148	142	
	CS’	1000	1000	230	230	95	33.2	CFRP	0.19	148	150	
	H1	1000	1000	150	150	95	118	HFRP	0.62	37.3	207	
	H2	1000	1000	150	150	89	35.8	HFRP	3.76	40.7	231	
	H2’	1000	1000	80	80	89	35.9	HFRP	3.76	40.7	171	
	H3	1000	1000	150	150	122	32.1	HFRP	1.22	44.8	237	
	H3’	1000	1000	80	80	122	32.1	HFRP	1.22	44.8	217	
2000	1	2000	2500	250	150	162	42	GFRP	0.28	85	622	[49]
	2	2000	2500	250	150	162	42	GFRP	0.28	85	698	
	3	2000	2500	250	150	162	42	GFRP	0.28	85	575	
	4	2000	2500	250	150	162	42	GFRP	0.28	85	534	
	5	2000	2500	250	150	162	42	GFRP	0.28	85	584	
2000	1	1800	3000	575	225	165	59	CFRP	0.57	147	1000	[50]
	2	1800	3000	575	225	165	59	CFRP	0.57	147	1200	
	3	1800	3000	575	225	165	59	CFRP	0.57	147	1328	
2000	1	2000	4000	500	250	138	35	GFRP	2.4	42	756	[51]
2003	SG1	2000	2000	200	200	142	33.3	GFRP	0.22	45	170	[23]
	SC1	2000	2000	200	200	142	34.7	CFRP	0.18	110	229	
	SG2	2000	2000	200	200	142	46.6	GFRP	0.47	45	271	
	SG3	2000	2000	200	200	142	30.3	GFRP	0.47	45	237	
	SC2	2000	2000	200	200	142	29.6	CFRP	0.43	110	317	
2003	GFR-1	2150	2150	250	250	120	29.5	GFRP	0.73	34	217	[25]
	GFR-2	2150	2150	250	250	120	28.9	GFRP	1.46	34	260	
	NEF-1	2150	2150	250	250	120	37.5	GFRP	0.87	28.4	206	
2003	ZJF5	1760	1760	250	250	75	45	CFRP-	1	100	234	[26]
2004	G-S1	1830	1830	250	250	100	40	GFRP	1.18	42	249	[52]
	G-S2	1830	1830	250	250	100	35	GFRP	1.05	42	218	
	G-S3	1830	1830	250	250	100	29	GFRP	1.67	42	240	
	G-S4	1830	1830	250	250	100	26	GFRP	0.95	42	210	
2005	1	2300	2000	635	250	175	27.6	GFRP	0.98	33	537	[27]
	2	2300	2000	635	250	175	27.6	GFRP	0.98	33	536	
	3	2300	2000	635	250	175	27.6	GFRP	0.95	33	531	
	7	2000	2000	635	250	175	27.6	GFRP	0.98	33	721	
	8	2000	2000	635	250	175	27.6	GFRP	0.98	33	897	
2005	G-S1	3000	2500	600	250	159	49.6	GFRP	1	44.6	740	[29]
	G-S2	3000	2500	600	250	159	44.3	GFRP	1.99	38.5	712	
	G-S3	3000	2500	600	250	159	49.2	GFRP	1.21	46.5	732	
	C-S1	3000	2500	600	250	165	49.6	CFRP	0.35	122.5	674	
	C-S2	3000	2500	600	250	165	44.3	CFRP	0.69	122.5	799	
2005	GS2	1830	1830	250	250	100	35	GFRP	1.05	42	218	[31]
	GSHS	1830	1830	250	250	100	71	GFRP	1.18	42	275	
2006	CS1	1900	1900	250	250	100	31	CFRP	0.41	120	251	[30]
	CS2	1900	1900	250	250	100	33	CFRP	0.54	120	293	
	CS3	1900	1900	250	250	100	25.7	CFRP	0.75	120	285	
	CSHD1	1900	1900	250	250	100	35.9	CFRP	0.54	120	325	
	CSHD2	1900	1900	250	250	100	38.6	CFRP	0.75	120	360	
	CSHS1	1900	1900	250	250	150	85.6	CFRP	0.36	120	399	
	CHSHS2	1900	1900	250	250	150	98.3	CFRP	0.5	120	446	
2007	1	1900	1900	250	250	110	70	GFRP	1	41	282	[53]
	2	1900	1900	250	250	110	70	GFRP	1.2	41	319	
	3	1900	1900	250	250	110	70	GFRP	1.5	41	384	
	4	1900	1900	250	250	160	70	GFRP	1.2	41	589	
	5	1900	1900	250	250	145	70	GFRP	1.2	41	487	
	6	1900	1900	250	250	135	70	GFRP	1.2	41	437	
2007	ZJEF1	1760	1000	250	250	120	25	CFRP	1.37	100	188	[54]
	ZJEF2	1760	1000	250	250	120	27	CFRP	0.94	100	156	
	ZJEF3	1760	1000	250	250	120	55	CFRP	1.37	100	211	
	ZJEF5	1760	1000	250	250	81	28	CFRP	1.37	100	97	
	ZJEF7	1760	1000	450	250	120	26	CFRP	1.37	100	196	
	ZJF8	1760	1760	350	250	101	28	CFRP	1.48	100	178	
	ZJF9	1760	1760	250	250	100	57.6	CFRP	1.48	100	272	
2007	G-S4	3000	2500	600	250	156	44.1	GFRP	1.2	44.5	707	[55]
	G-S5	3000	2500	600	250	156	44.1	GFRP	1.2	44.5	735	
2008	F1	1200	1200	200	200	82	37.4	GFRP	1.1	46	165	[56]
	F2	1200	1200	200	200	112	33	GFRP	0.81	46	170	
	F3	1200	1200	200	200	82	38.2	GFRP	1.29	46	210	
	F4	1200	1200	200	200	82	39.7	GFRP	1.54	46	230	
2009	GFU1	2300	2300	225	225	110	36.3	GFRP	1.17	48.2	222	[57]
	GFB2	2300	2300	225	225	110	36.3	GFRP	2.14	48.2	246	
	GFB3	2300	2300	225	225	110	36.3	GFRP	3	48.2	248	
	GFBF3	2300	2300	225	225	110	33.8	GFRP	3	48.2	330	
2010	S3	1500	1500	150	150	135	33.5	BFRP	0.29	100	145	[58]
	S4	1500	1500	150	150	135	35.6	BFRP	0.55	100	275	
	S5	1500	1500	150	150	135	32.8	BFRP	0.42	100	235	
	S6	1500	1500	150	150	135	32.5	BFRP	0.42	100	225	
	S7	1500	1500	150	150	135	22.6	BFRP	0.42	100	170	
	S8	1500	1500	150	150	135	41.8	BFRP	0.42	100	235	
	S9	1500	1500	150	150	135	40.6	BFRP	0.42	100	200	
2010	NC-G-45	300	300	25	25	45	47.8	GFRP	0.78	76	44	[59]
	NC-G-0/90	300	300	25	25	45	47.8	GFRP	0.78	76	45	
	NC-C-45	300	300	25	25	45	47.8	CFRP	0.24	230	39	
	NC-C-0/90	300	300	25	25	45	47.8	CFRP	0.24	230	45	
	SFRC-C-45	300	300	25	25	45	47.8	CFRP	0.24	230	63	
	UHPC-C-45	300	300	25	25	45	179	CFRP	0.24	230	97	
	UHPC-C-0/90	300	300	25	25	45	179	CFRP	0.24	230	98	
2010	A	1500	1500	150	150	130	22.16	GFRP	0.42	45.6	176	[60]
	B-2	1500	1500	150	150	130	32.46	GFRP	0.42	45.6	209	
	B-3	1500	1500	150	150	130	32.4	GFRP	0.55	45.6	245	
	B-4	1500	1500	150	150	130	32.8	GFRP	0.29	45.6	167	
	B-5	1500	1500	150	150	130	33.2	GFRP	0.42	45.6	217	
	B-6	1500	1500	150	150	130	28.32	GFRP	0.42	45.6	222	
	B-7	1500	1500	150	150	130	46.05	GFRP	0.42	45.6	253	
2011	G200n	3000	2500	600	250	155	49.1	GFRP	1.20	43	732	[61]
	G175N	3000	2000	600	250	135	35.2	GFRP	1.20	43	484	
	G150N	3000	2000	600	250	110	35.2	GFRP	1.20	43	362	
	G175h	3000	2000	600	250	135	64.8	GFRP	1.20	43	704	
	G175n0.7	3000	2000	600	250	135	53.1	GFRP	0.7	43	549	
	G175n0.35	3000	2000	600	250	137	53.1	GFRP	0.35	43	506	
	C175N	3000	2000	600	250	140	40.3	GFRP	0.40	122	530	
2012	A	1500	1500	150	150	130	22.2	GFRP	0.42	45.6	176	[62]
	B-2	1500	1500	150	150	130	32.5	GFRP	0.42	45.6	209	
	B-3	1500	1500	150	150	130	32.4	GFRP	0.55	45.6	245	
	B-4	1500	1500	150	150	130	32.8	GFRP	0.29	45.6	167	
	C	1500	1500	150	150	130	44.4	GFRP	0.42	45.6	252	
2013	GSL-PUNC-0.4	2200	2200	200	200	130	48.8	GFRP	0.48	48	180	[34]
	GSL-PUNC-0.5	2200	2200	200	200	130	48.8	GFRP	0.68	48	212	
	GSL-PUNC-0.6	2200	2200	200	200	130	48.8	GFRP	0.92	48	244	
2013	G (0.7) 30/20	2500	2500	300	300	134	34.3	GFRP	0.71	48.2	329	[35]
	G (1.6) 30/20	2500	2500	300	300	131	38.6	GFRP	1.56	48.1	431	
	G (1.6) 30/20-H	2500	2500	300	300	131	75.8	GFRP	1.56	57.4	547	
	G (1.2) 30/20	2500	2500	300	300	131	37.5	GFRP	1.21	64.9	438	
	G (0.3) 30/35	2500	2500	300	300	284	34.3	GFRP	0.34	48.2	825	
	G (0.7) 30/35	2500	2500	300	300	284	39.4	GFRP	0.73	48.1	1071	
	G (1.6) 30/35	2500	2500	300	300	275	38.2	GFRP	1.61	56.7	1492	
	G (1.6) 30/35-H	2500	2500	300	300	275	75.8	GFRP	1.61	56.7	1600	
	G(0.7) 30/20-B	2500	2500	300	300	134	38.6	GFRP	0.71	48.2	386	
	G(0.7) 45/20	2500	2500	300	300	134	44.9	GFRP	0.71	48.2	400	
	G (1.6) 45/20-B	2500	2500	300	300	131	39.4	GFRP	1.56	48.1	511	
	G (0.3) 30/35-B	2500	2500	300	300	284	39.4	GFRP	0.34	48.2	781	
	G (0.7) 30/35-B-2	2500	2500	300	300	281	46.7	GFRP	0.73	48.1	1195	
	G (0.3) 45/35	2500	2500	300	300	284	48.6	GFRP	0.34	48.2	911	
	G (1.6) 30/20-B	2500	2500	300	300	131	32.4	GFRP	1.56	48.1	451	
	G (1.6) 45/20	2500	2500	300	300	131	32.4	GFRP	1.56	48.1	504	
	G (0.7) 30/35-B-1	2500	2500	300	300	181	29.6	GFRP	0.73	48.1	1027	
	G(0.3) 45/35-B	2500	2500	300	300	284	32.4	GFRP	0.34	48.2	1020	
	G (0.7) 45/35	2500	2500	300	300	281	29.6	GFRP	0.73	48.1	1248	
2015	GSC-0.9-XX-0.4	2800	1500	300	300	160	41	GFRP	0.9	60.505	251	[63]
	GSC-1.35-XX-0.4	2800	1500	300	300	160	41	GFRP	1.35	60.505	268	
	GSC-1.8-XX-0.4	2800	1500	300	300	160	41	GFRP	1.7	60.505	277	
	GSC-0.9-XX-0.2	2800	1500	300	300	160	41	GFRP	0.85	60.505	239	
	GSC-0.9-XX-0.3	2800	1500	300	300	160	41	GFRP	0.9	60.505	159	
	GRD-0.9-XX-0.4	2800	1500	300	300	160	41	GFRP	0.9	59.877	191	
2015	G-0.6%-12-125 T&B	1425	500	500	25	119	68.1	GFRP	0.6	67.4	344	[64]
	G-0.6%-16-300 T&B	1425	500	500	25	117	65.7	GFRP	0.6	67.4	365	
	B-0.6%-12-125 T&B	1425	500	500	25	119	69.3	BFRP	0.6	54	300	
	B-0.6%-16-300 T&B	1425	500	500	25	117	66.1	BFRP	0.6	54	295	
2016	GSC-0.9-XX-0.4	2600	1450	300	300	160	81	GFRP	0.87	60.505	251	[65]
	GSC-1.35-XX-0.5	2600	1450	300	300	160	85	GFRP	1.28	60.505	272	
	GSC-1.8-XX-0.4	2600	1450	300	300	160	80	GFRP	1.7	60.505	288	
2016	S2-B	3000	2000	600	250	160	48.81	BFRP	0.8	69.3	548	[66]
	S3-B	3000	2000	600	250	160	42.2	BFRP	0.79	69.3	665	
	S4-B	3000	2000	600	250	160	42.2	BFRP	0.8	69.3	566	
	S5-B	3000	2000	600	250	160	47.9	BFRP	1.2	69.3	716	
	S6-B	3000	2000	600	250	160	47.9	BFRP	0.4	69.3	576	
	S7-B	3000	2000	600	250	160	47.9	BFRP	0.4	69.3	436	
2016	GN-0.65	2600	2600	300	300	160	42	GFRP	0.65	69.3	363	[67]
	GN-0.98	2600	2600	300	300	160	38	GFRP	0.98	68	378	
	GN-1.30	2600	2600	300	300	160	39	GFRP	1.3	68	425	
	GH-0.65	2600	2600	300	300	160	70	GFRP	0.65	68	380	
	G-00-XX	2800	2800	300	300	160	38	GFRP	0.65	68	421	[68]
2016	G-15-XX	2800	2800	300	300	160	42	GFRP	0.65	68	363	
	G-30-XX	2800	2800	300	300	160	42	GFRP	0.65	68	296	
	R-15-XX	2800	2800	300	300	160	40	GFRP	0.65	68	320	
2017	NW59	800	800	250	250	176	59	GFRP	0.703	68	719	[69]
2017	SG1	1100	1100	150	150	62	29.8	GFRP	0.22	47	136	[70]
	SO1	1100	1100	150	150	62	37.3	GFRP	0.13	47	68	
	SO2	1100	1100	150	150	62	32.6	GFRP	0.13	47	85	
	SO3	1100	1100	150	150	62	30.5	GFRP	0.22	47	80	
	SO4	1100	1100	150	150	62	35.4	GFRP	0.22	47	100	
	SO5	1100	1100	150	150	62	30.1	GFRP	0.22	47	102	
2018	GFS1	3000	2200	200	200	180	36.7	GFRP	1.57	47	410	[71]
	GFS2	3000	2200	200	200	180	36.7	GFRP	1.2	47	360	
	GFS3	3000	2200	200	200	180	36.7	GFRP	0.79	47	370	
2018	H-1.0-XX	2800	2800	300	300	160	80	GFRP	0.98	65	461	[72]
	H-1.5-XX	2800	2800	300	300	160	84	GFRP	1.46	65	541	
	H-2.0-XX	2800	2800	300	300	160	87	GFRP	1.93	65	604	
2019	C-F-S-10-4	600	600	100	100	80	51	CFRP	0.3	144	103	[39]
	C-F-S-10-6	600	600	100	100	80	52	CFRP	0.45	144	127	
	S-F-D-10-4	600	600	100	100	80	46	CFRP	0.6	144	112	
	S-F-D-10-6	600	600	100	100	80	60	CFRP	0.9	144	129	
	S-F-S-10-4	600	600	100	100	80	52	CFRP	0.3	144	79	
	S-F-S-10-6	600	600	100	100	80	48	CFRP	0.45	144	107	
	S-F-S-7.5-4	600	600	100	100	60	49	CFRP	0.41	144	57	
	S-F-S-7.5-6	600	600	100	100	60	49	CFRP	0.61	144	79	
2019	G	2500	1350	300	300	160	41.4	GFRP	1.55	65	314	[73]
2019	G1 (1.06)	2500	2500	300	300	151	52	GFRP	1.06	62.6	140	[74]
	G2 (1.51)	2500	2500	300	300	151	92	GFRP	1.51	62.6	140	
	G3(1.06)-SL	2500	2500	300	300	151	45	GFRP	1.06	62.6	180	
2020	A30-1	1500	1500	300	300	88	27.4	GFRP	1.28	51.1	191	[75]
	A30-2	1500	1500	300	300	108	27.3	GFRP	1.05	51.1	289	
	A30-3	1500	1500	300	300	138	26.2	GFRP	0.82	51.1	413	
	A30-4	1500	1500	350	350	86	26.8	GFRP	1.31	51.1	209	
	A40-1	1500	1500	350	350	88	28.2	GFRP	1.28	51.1	232	
	A40-2	1500	1500	350	350	88	26.4	GFRP	0.89	51.1	221	
	A40-3	1500	1500	300	300	88	28.6	GFRP	1.28	51.1	236	
	A50-1	1500	1500	300	300	88	29.2	GFRP	1.28	51.1	253	
	A50-2	1500	1500	300	300	90	32.2	GFRP	0.87	54.1	237	
	A50-3	1500	1500	350	350	88	26.7	GFRP	1.28	51.1	280	
2020	S40-1	1500	1500	300	300	88	32.3	GFRP	0.98	51.1	314	[76]
	S50-1	1500	1500	300	300	86	43.2	GFRP	0.7	54.4	187	
2020	G4(1.06)-H	2500	2500	300	300	151	92	GFRP	1.06	62.6	134	[77]
2020	F1	1600	1600	200	200	125	24.97	CFRP	0.89	123	262	[78]
2021	G-N-0.3	2500	1300	300	300	160	37.1	GFRP	1.04	65	260	[79]
	G-H-0.3	3000	2200	200	200	160	85.8	GFRP	1.04	65	306	
	G-N-0.6	3000	2200	200	200	160	38.8	GFRP	1.04	65	178	
	G-H-0.6	3000	2200	200	200	160	86	GFRP	1.04	65	213	
2021	0F-605	2000	2000	250	250	125	38.2	GFRP	2.81	50.6	463	[80]
	0F-80F	2000	2000	250	250	125	38.2	GFRP	2.11	50.6	486	
	0F-1105	2000	2000	250	250	125	38.2	GFRP	1.53	50.6	436	
	1.25F-60S	2000	2000	250	250	125	38.2	GFRP	2.81	50.6	455	
	1.25F-80S	2000	2000	250	250	125	38.2	GFRP	2.11	50.6	506	
	1.25F-110S	2000	2000	250	250	125	38.2	GFRP	1.53	50.6	498	
2022	SA1	500	500	55	55	36	45	BFRP	0.84	50	30	[81]
	SA2	500	500	55	55	36	45	GFRP	0.84	42	28	
	SA4	500	500	55	55	36	45	BFRP	0.56	50	26	
	SA5	500	500	55	55	36	45	GFRP	0.56	42	24	
	SA7	500	500	55	55	36	65	BFRP	0.84	50	35	
	SA0	500	500	55	55	36	45	BFRP	0.84	50	28	
2022	CFRP1	1670	1670	1075	1075	52	29.62	CFRP	0.36	140	169	[82]
	CFRP2	1670	1670	1075	1075	52	34.59	CFRP	0.36	140	178	
	CFRP3	1670	1670	1075	1075	52	34.59	CFRP	0.36	140	208	
	BFRP1	1670	1670	1075	1075	52	29.62	BFRP	0.36	55	103	
	BFRP2	1670	1670	1075	1075	52	34.59	BFRP	0.36	55	120	
	BFRP3	1670	1670	1075	1075	52	34.59	BFRP	0.36	55	144	
	Minimum	300	300	25	25	36	22.16		0.13	28.4	24.34	
	Maximum	3000	4000	1075	1075	284	179		3.76	230	1600	
	Mean	1915	1715	303	235	125	44		1	75	372	
	Variation	40%	39%	66%	65%	40%	44%		64%	55%	82%	

**Table 3:** Statistical measures for all strength models.

Design Model	R^2^	RMSE	MAE	Mean	C.O.V.	Lower 95%	Maximum	Minimum
G	0.67	205	144	0.82	0.38	0.78	1.85	0.15
JSCE	0.69	337	238	2.71	0.38	2.58	8.08	0.69
Gd	0.69	776	653	0.36	0.37	0.34	0.78	0.08
MT	0.69	183	121	1.18	0.36	1.13	2.92	0.23
O	0.71	170	110	1.00	0.38	0.95	3.01	0.18
Z	0.67	200	126	0.94	0.38	0.90	2.81	0.16
Jb	0.67	182	110	1.15	0.38	1.10	2.59	0.21
ACI	0.69	272	194	2.18	0.45	2.06	6.90	0.36
Eg	0.68	222	137	0.86	0.37	0.82	2.59	0.13
Zg	0.70	166	106	1.00	0.35	0.96	2.43	0.19
TS	0.70	255	176	1.78	0.36	1.70	3.96	0.33
CSA	0.72	165	110	1.19	0.40	1.13	3.17	0.21
NR	0.56	360	252	0.63	0.45	0.60	1.60	0.14
H	0.70	290	203	2.16	0.36	2.07	5.56	0.40
KS	0.71	163	103	1.07	0.37	1.02	3.07	0.19
CCCM	0.67	198	126	1.06	0.44	1.00	3.48	0.22
Hz	0.72	164	105	1.00	0.45	0.95	3.54	0.19
EE-a	0.67	305	195	0.74	0.38	0.70	2.27	0.11
EE-b	0.70	233	160	1.61	0.35	1.54	4.24	0.30
Ju	0.70	173	117	1.26	0.36	1.20	3.60	0.22
A	0.71	163	106	1.13	0.36	1.07	3.21	0.20

## Data Availability

All data are available within the manuscript.
